# Nephrotic syndrome with focal segmental glomerular lesions unclassified by Columbia classification; Pathology and clinical implication

**DOI:** 10.1371/journal.pone.0244677

**Published:** 2021-01-05

**Authors:** Takaya Ozeki, Michio Nagata, Takayuki Katsuno, Koji Inagaki, Kazunori Goto, Sawako Kato, Yoshinari Yasuda, Naotake Tsuboi, Shoichi Maruyama

**Affiliations:** 1 Department of Nephrology, Nagoya University Graduate School of Medicine, Nagoya, Japan; 2 Faculty of Medicine, Kidney and Vascular Pathology, University of Tsukuba, Tsukuba, Japan; 3 Department of Nephrology, Chutoen General Medical Center, Kakegawa, Japan; Istituto Di Ricerche Farmacologiche Mario Negri, ITALY

## Abstract

**Background:**

The Columbia classification is widely used for diagnosis of focal segmental glomerulosclerosis (FSGS). In practice, we occasionally encounter segmental glomerular lesions unclassified as Columbia classification. We analyzed the clinical implication of unclassified segmental lesions comparing with Columbia-classified FSGS.

**Methods:**

A retrospective cohort study from 13 local hospitals in Japan. From 172 biopsy cases diagnosed with FSGS or minimal change disease (MCD)/FSGS spectrum with unclassified segmental lesions, adult patients with nephrotic syndrome who received immunosuppressive therapies were included. The cases are classified by pathology, i.e., typical FSGS lesions sufficiently classified into subgroups of Columbia classification: collapsing (COL), tip (TIP), cellular (CEL), perihilar (PH), and not otherwise specified (NOS), and unclassified by the Columbia classification into three subgroups: “endothelial damage,”; “simple attachment,”; and “minor cellular lesion,”. The response to immunosuppressive treatment and 30% decline of eGFR were compared.

**Results:**

Among 48 eligible cases, all were Japanese, 34 were typical FSGS; 13 TIP, 15 CEL, 6 NOS, and no COL or PH cases. Fourteen were unclassified cases: endothelial damage (n = 6), simple attachment (n = 5), and minor cellular lesion (n = 3). The median age of overall patients was 60 years old and the median of eGFR and urinary protein creatinine ratio was 51.5 mL/min/1.73m^2^ and 7.35, respectively. They received similar therapeutic regimen. Kaplan-Meier analysis revealed no significant difference in treatment response between typical FSGS and unclassified cases. Evaluating among the subgroups, endothelial damage, simple attachment and minor cellular lesion showed similar treatment response to TIP or CEL. No significant difference was also observed in the 30% decline of eGFR.

**Conclusions:**

Japanese adult patients with nephrotic syndrome showing unclassified segmental lesions as Columbia classification may be equivalent clinical impact as Columbia classification of FSGS.

## Introduction

Focal segmental glomerulosclerosis (FSGS) is a major cause of nephrotic syndrome (NS) leading to end stage renal disease [1]. FSGS was classically indicated in pediatric patients with steroid-resistant NS who demonstrated segmental obliteration of the glomerular capillaries [2]. Since the 1980s, non-classical segmental lesions, including glomerular tip lesions, cellular lesions, and collapsing nephropathy, have also been identified as FSGS [3–5]. To date, these pathological features are considered to represent the consequences of glomerular injury centered on podocytes and their repair processes [6,7]. In 2004, the Columbia classification was proposed for the pathological evaluation of FSGS [8]. The Columbia classification is composed of five morphological subgroups: Collapsing variant (COL), tip variant (TIP), cellular variant (CEL), perihilar variant (PH), and not otherwise specified (NOS). Because the variants are classified by morphological patterns that are relevant to the clinical features, this classification is widely used; indeed, several studies have confirmed its utility for clinical outcomes, particularly good course in TIP, and bad in COL [9–15].

By a simple concept that allocates each patient to one typical light microscopic pattern with hierarchical prioritization, the Columbia classification is well accepted. However, this concept can cause equivocal judgement in practice, because the segmental lesions do not always fulfill the definition of Columbia variants (we call unclassified lesions). We do not know whether these unclassified lesions are presumptive for typical FSGS lesions or their clinical relevance. There have been no reports focusing on such unclassified segmental lesions.

The main purpose of this study was to evaluate the clinical implication of specific segmental glomerular lesions that are not typically included in the Columbia classification.

## Materials and methods

This was a retrospective multicenter cohort study that involved Nagoya University and its 12 affiliated hospitals: Anjo Kosei Hospital, Ichinomiya Municipal Hospital, Ogaki Municipal Hospital, Kasugai Municipal Hospital, Gifu Prefectural Tajimi Hospital, Konan Kosei Hospital, Chubu Rosai Hospital, Toyohashi Municipal Hospital, Handa City Hospital, Japanese Red Cross Nagoya Daiichi Hospital, Nagoya Kyoritsu Hospital, and Yokkaichi Municipal Hospital.

### Patients

**Fig 1:**
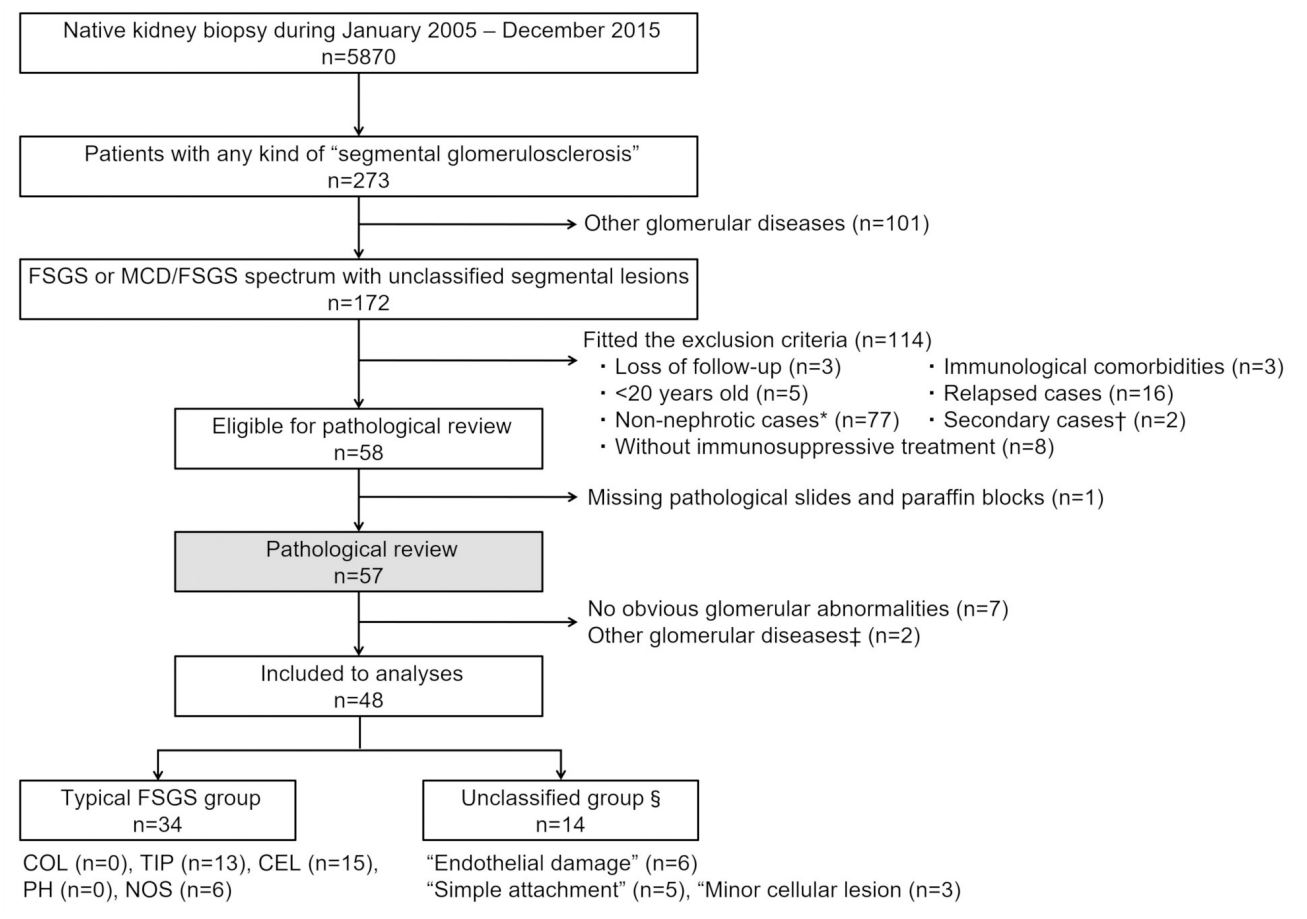
Flow diagram of patient selection in the present study. Among 172 patients who were diagnosed with FSGS or minor glomerular abnormality with segmental glomerular lesions, adult cases with first episode of nephrotic syndrome who received immunosuppressive treatment were eligible for pathological review. The pathological review classified the patients into two groups: Typical FSGS, with typical segmental lesions according to the Columbia classification, and unclassified group, without such lesions. Patients in the typical FSGS group were categorized into five subgroups of histopathological variants of the Columbia classification. Patients in the unclassified group were categorized into three subgroups: “endothelial damage”, “simple attachment”, and “minor cellular lesion”. * Non-nephrotic cases included 26 patients whose etiologies were recorded: obesity (n = 15), hypertension (n = 7), unilateral kidney (n = 2), low birth weight (n = 1) and vesicoureteral reflux (n = 1). † Secondary cases: HIV infection (n = 1), genetic mutation (n = 1). ‡ Other glomerular diseases in pathological review: Nephrosclerosis (n = 1) and mesangial proliferative glomerulonephritis (n = 1). § Definitions of unclassified group are shown in Fig 2. Abbreviations: FSGS, Focal segmental glomerulosclerosis; NS, Nephrotic syndrome; COL, Collapsing variant; TIP, Tip variant; CEL, Cellular variant; PH, Perihilar variant; NOS, Not otherwise specified.

All the patients underwent kidney biopsy from January 2005 to December 2015 and pathological diagnoses were initially made in the department of nephrology of Nagoya University. As shown in Fig 1, among total 5870 native kidney biopsy cases, 273 patient who had any kind of “segmental glomerulosclerosis” were extracted. After excluding other specific glomerular diseases that were diagnosed by serological or pathological findings including immunofluorescence and electron microscopy such as IgA nephropathy, membranous nephropathy, lupus nephritis and diabetic nephropathy, 172 patients who were recorded as FSGS or minimal change disease (MCD)/FSGS spectrum with unclassified segmental lesions were included. Of 172 patients, we included the patients who fulfilled the following conditions: (i) ≥ 20 years old, (ii) first episode of NS, (iii) demonstrated NS at the initiation of immunosuppressive therapy (serum albumin < 3.0 g/dL and urinary protein level > 3.5 g/gCr), and (iv) received immunosuppressive treatment such as glucocorticoid and/or other kind of immunosuppressive agents. Patients who lacked follow-up data, who had concomitant disorders that required immunosuppressive therapies, < 20 years old, who underwent biopsy for relapses of NS, non-nephrotic cases, secondary FSGS (e.g., patients with HIV infection or genetic mutation), or cases without immunosuppressive treatment were excluded. For pathological review, patients whose pathological slides or paraffin blocks were unavailable were also excluded.

### Pathological review

Slide preparation and storage of paraffin blocks were carried out in Nagoya University, with the exception of one case. In the present study, all pathological slides were newly reviewed by one renal pathologist outside of Nagoya University (MN) without knowing the clinical outcomes. All specimens had at least 12 serial sections stained with Periodic acid Schiff stain, Periodic acid methenamine silver stain, Masson trichrome stains, and Hematoxylin-eosin stain. If the slides contained less than 5 glomeruli, or no evident findings on FSGS, additional sections were obtained from the paraffin blocks (up to 32 sections). Patients who had no detectable abnormalities even after evaluating additional sections were excluded from the analyses. The total number of glomeruli and the number/percentage of glomeruli with global sclerosis and segmental lesions (see below) were counted. The degree of interstitial fibrosis and tubular atrophy (IF/TA) was also evaluated.

#### Typical FSGS group

Pathological review for segmental glomerular lesions was conducted based on the Columbia classification [8]. Patients with typical FSGS lesions were classified into following subgroups: COL, TIP, CEL, PH, or NOS.

#### Unclassified group and its definition

If the patients who only had abnormal findings that did not fit the definition of any variants in the Columbia classification despite the evaluation of additional sections, they were categorized into the “unclassified group” and divided into three subgroups according to the characteristics of the finding as follows. All the patients did not have any specific findings of immune deposits on glomeruli in immunofluorescence and electron microscopy.

#### (1) “Endothelial damage”

**Fig 2:**
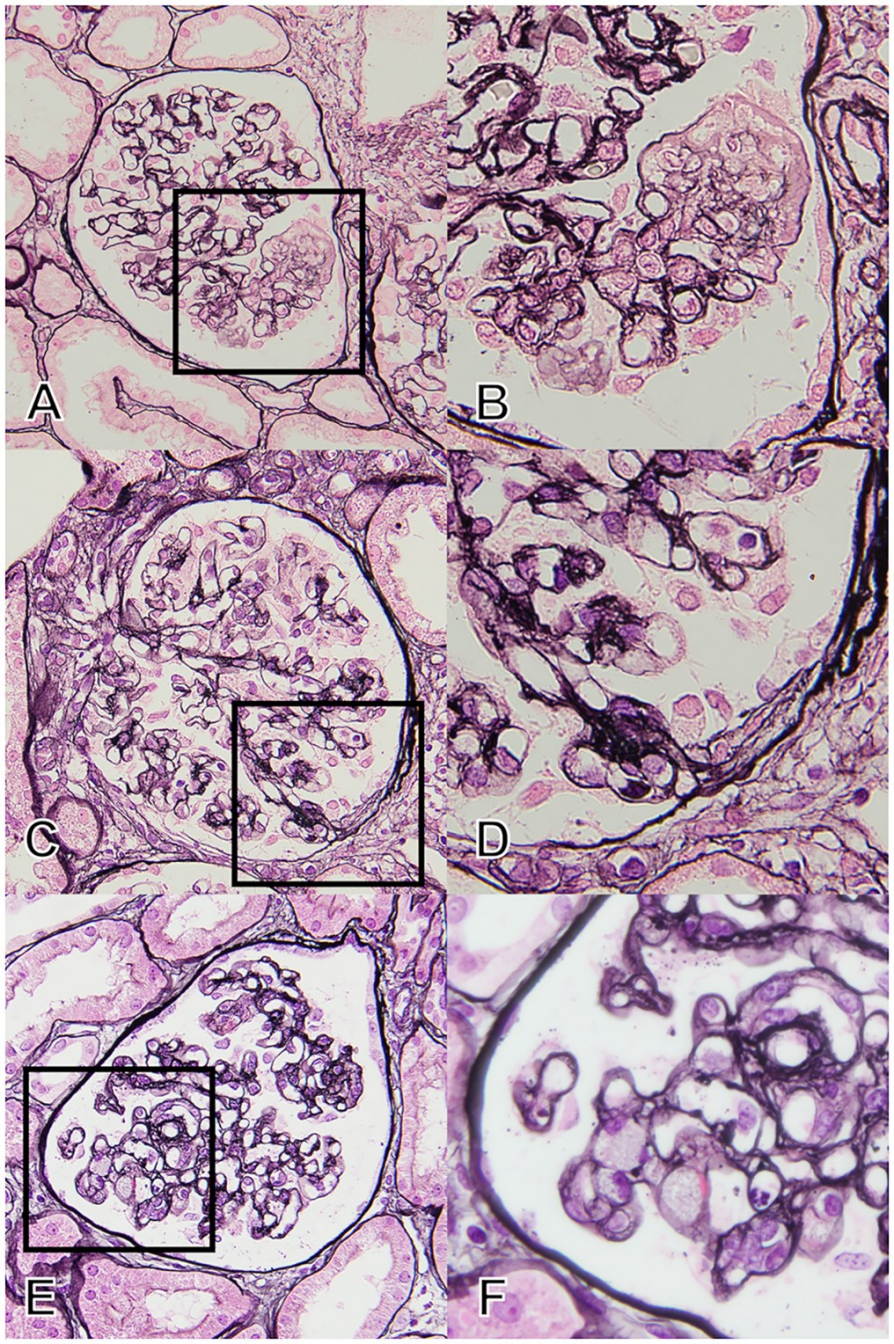
Pathological findings in patients who did not fit any of the FSGS variants in the Columbia classification: The unclassified group. (A and B) Typical finding of “endothelial damage” by PAM staining. The patients had findings suggesting endothelial damage, i.e., mesangiolysis and/or double contour of the glomerular basement membrane without endocapillary occlusion or matrix accumulation. (C and D) The finding of “simple attachment” by PAM staining. The image indicates small fibrous synechia to the Bowman capsule without any endocapillary or extracapillary interactions. The patients had no other findings on FSGS. (E and F) The finding of “minor cellular lesion” by PAM staining. The image indicates cellular occlusion of the glomerular capillaries by foam cell(s), but with a distribution of less than 25% of the tuft. The patients had no other findings on FSGS. A, C, and E are at ×400 magnification with PAM staining, and B, D, and F are at ×1000 magnification with PAM staining. Abbreviations: FSGS, Focal segmental glomerulosclerosis; PAM, Periodic acid methenamine silver stain.

The patients in this subgroup had findings that suggested endothelial damage: Segmental expansion of the glomerular tuft with mesangiolysis, and/or double contour of the glomerular basement membrane (GBM) without typical FSGS lesions (Fig 2A and 2B and S1A–S1D Fig).

#### (2) “Simple attachment”

In this subgroup, the term of “simple attachment” indicates a fibrous adhesion between the tuft and Bowman’s capsule without typical extracapillary/endocapillary cellular proliferation or matrix accumulation as defined in the Oxford Classification for IgA nephropathy [16] (Fig 2C and 2D and S1E–S1H Fig).

#### (3) “Minor cellular lesion”

We defined “minor cellular lesion” as the endocapillary obstruction by foam cell(s) limited to less than 25% of the tuft (Fig 2E and 2F and S1I–S1L Fig).

### Collection of clinical data

The clinical data were collected retrospectively from medical records independent of pathological review. The clinical course of the patients was followed until December 2017. The initiation of immunosuppressive treatment was defined as time of baseline. The following baseline information was recorded: Age, sex, body mass index, blood pressure, and prescription of antihypertensive drugs. Laboratory data at the baseline were also collected: Serum total protein, albumin, total cholesterol, and creatinine (Scr) concentration. The estimated glomerular filtration rate (eGFR) was obtained using the equation for Japanese patients: eGFR [mL/min/1.73 m^2^] = 194 × Scr^-1.094^ × Age^-0.287^ × 0.739 (if female) [17]. The urinary occult blood level, and urinary protein-creatinine ratio (g/gCr) or 24-hour urinary protein excretion (g/day) were also measured. Data regarding the initial treatment: Initial prednisolone dose, methylprednisolone pulse, combined other immunosuppressive agents, 25% human albumin administration, temporary hemodialysis, and low-density lipoprotein apheresis, and induction of remission were collected.

### Outcomes

The primary outcomes of interest were as follows: (i) Attainment of complete remission (CR) of proteinuria and (ii) the incidence of renal event: 30% decline of eGFR and maintenance dialysis. CR was defined as the reduction of proteinuria to < 0.3 g/day or < 0.3 g/gCr in the present study. For evaluation of the 30% decline in eGFR, the basis of the eGFR decline during the entire observation was obtained from the minimal value of serum creatinine among those of at the initiation of immunosuppressive treatment (baseline), and after 1, 2, 6, and 12 months from the baseline considering the recovery of renal function after acute kidney injury [18]. As secondary outcome, the incidence of partial remission, maintenance dialysis and transplantation were also evaluated. Partial remission was defined according to the clinical guidelines for nephrotic syndrome by Japanese Society of Nephrology [19]: Partial remission, reduction of proteinuria to < 3.5 g/day or < 3.5 g/gCr; Non-response, failed to reduce urine protein less than 3.5 g/day or 3.5 g/gCr.

### Statistics

The clinical characteristics of the patients were expressed as medians/interquartile ranges (IQR) and as frequency number/percentages. The Kruskal-Wallis test was used to compare the continuous variables, and the Chi-squared test was used to compare the proportions of categorical variables among the pathological subgroups. Kaplan–Meier analysis and the log-rank test were used to compare the clinical course between the typical FSGS group and the unclassified group or among the subgroups. Patients who dropped out, started renal replacement therapies such as maintenance dialysis or kidney transplantation, or died before the end of the follow-up period were considered as censoring. We did not use either a univariate or multivariate Cox proportional hazard model because of the small number of patients in each subgroup. Statistical analyses were conducted using STATA IC version 14.0 (StataCorp LLC, College Station, TX, USA). The statistical significance level was set at P < 0.05.

### Ethics

Written informed consent was obtained from all participants, and the study was approved by the ethics committee of Nagoya University (approval number: 2010-1135-4) in agreement with the Declaration of Helsinki.

## Results

In total, 48 patients were eligible for analyses (Fig 1), all of whom were Japanese. The mean number of serial slices for pathological review was 14.5. Of 48 patients, 34 (70.8%) were typical FSGS: 13 patients (27.1%) were TIP, 15 patients (31.3%) were CEL, 6 patients (12.5%) were NOS, and no patients were COL or PH. Fourteen patients (29.2%) did not fit any variant of the Columbia classification (unclassified group): The subgroups “endothelial damage”, “simple attachment”, and “minor cellular lesion” had 6, 5, and 3 patients, respectively. In the unclassified group, no patient showed findings that were applicable to more than one subgroup.

### Clinical characteristics of each pathological subgroup

The clinical characteristics of the patients comparing between the typical FSGS group and the unclassified group and among the subgroups are summarized in S1 Table and Table 1, respectively. The patients with NOS were relatively young, while the patients with CEL and minor cellular lesion showed a lower eGFR at the initiation of immunosuppressive treatment. No remarkable differences were observed in the other clinical parameters among the subgroups.

**Table 1:** Baseline characteristics of the subgroups.

	Overall (n = 48)	Typical FSGS group			Unclassified group			P-value
		TIP (n = 13)	CEL (n = 15)	NOS (n = 6)	Endothelial damage (n = 6)	Simple attachment (n = 5)	Minor cellular lesion (n = 3)	
**Patient characteristics**								
Age	60 [44–67]	50 [39–59]	63 [37–72]	45 [28–55]	59 [52–70]	64 [63–71]	78 [65–79]	0.021
Sex, male	33 (68.8)	10 (76.9)	11 (73.3)	4 (66.7)	2 (33.3)	5 (100.0)	1 (33.3)	0.151
Height, cm	164 [157–170]	166 [163–170]	167 [157–170]	163.7 [150–164]	157.4 [153–160]	165 [165–165]	155 [155–159]	0.23
Body weight, kg	62.3 [55.0–71.5]	62.4 [56.2–71.7]	66.1 [58.9–72.3]	62.1 [44.5–70.4]	54.3 [49.0–57.6]	62.9 [62.4–71.4]	58.9 [42.6–61.2]	0.29
Body mass index	23.5 [21.3–26.0]	23.5 [22.3–26.5]	23.5 [21.8–26.3]	23.2 [19.8–26.2]	21.3 [20.3–24.6]	23.1 [23.1–23.9]	24.5 [16.9–25.5]	0.82
Systolic BP, mmHg	129 [121–149]	142 [122–151]	141 [122–150]	132 [103–148]	120 [113–124]	121 [119–126]	144 [141–146]	0.169
Diastolic BP, mmHg	80 [71–88]	84 [75–90]	77 [70–86]	79 [70–98]	80 [75–82]	68 [60–87]	68 [56–88]	0.47
Diabetes mellitus	2 (4.2)	0 (0.0)	0 (0.0)	1 (16.7)	0 (0.0)	1 (20.0)	0 (0.0)	0.21
Hypertension	19 (39.6)	3 (23.1)	9 (60.0)	2 (33.3)	1 (16.7)	2 (40.0)	2 (66.7)	0.27
ACE-I/ARB	19 (39.6)	4 (30.8)	7 (46.7)	2 (33.3)	2 (33.3)	3 (60.0)	1 (33.3)	0.87
Diuretics	30 (62.5)	8 (61.5)	10 (66.7)	2 (33.3)	4 (66.7)	3 (60.0)	3 (100.0)	0.53
Statins	22 (45.8)	5 (38.5)	7 (46.7)	3 (50.0)	3 (50.0)	4 (80.0)	0 (0.0)	0.39
**Laboratory data**								
Total protein, g/dL	4.6 [3.7–5.0]	4.2 [4.0–5.1]	4.6 [3.7–4.9]	4.3 [3.3–4.7]	5.3 [5.0–5.4]	3.7 [3.7–4.8]	4.2 [3.8–5.7]	0.39
Albumin, g/dL	1.9 [1.5–2.3]	1.97 [1.8–2.3]	1.6 [1.1–1.9]	1.8 [1.1–2.3]	2.3 [1.7–2.4]	1.5 [1.2–2.1]	2.1 [2.1–3.0]	0.24
Creatinine, mg/dL	1.02 [0.85–1.60]	1.01 [0.85–1.40]	1.5 [1.01–2.04]	0.87 [0.67–1.09]	0.77 [0.63–0.91]	1.17 [0.89–1.44]	1.6 [0.64–1.90]	0.016
eGFR, mL/min/1.73m2	51.5 [38.0–69.7]	56.9 [41.7–69.4]	39 [22.6–50.6]	72.6 [51.8–104.3]	72.4 [57.3–79.7]	48.1 [38.2–66.8]	27.5 [25.9–66.7]	0.037
Temporary dialysis	5 (10.4)	1 (7.7)	3 (20.0)	0 (0.0)	0 (0.0)	1 (20.0)	0 (0.0)	0.58
Urinary protein, g/gCr	7.35 [5.06–8.98]	5.5 [4.82–9.67]	7.4 [5.32–9.41]	8.13 [5.06–8.98]	5.62 [4.48–7.79]	7.49 [6.46–8.26]	5.55 [5.51–8.00]	0.73
Urinary occult blood								
(-)	4 (8.5)	0 (0.0)	2 (13.3)	1 (16.7)	0 (0.0)	1 (25.0)	0 (0.0)	0.68
(+/-)	2 (4.3)	1 (7.7)	0 (0.0)	0 (0.0)	1 (16.7)	0 (0.0)	0 (0.0)	
(1+)	15 (31.9)	5 (38.5)	6 (40.0)	1 (16.7)	1 (16.7)	2 (50.0)	0 (0.0)	
(2+)	15 (31.9)	4 (30.8)	5 (33.3)	3 (50.0)	1 (16.7)	0 (0.0)	2 (66.7)	
(3+)	11 (23.4)	3 (23.1)	2 (13.3)	1 (16.7)	3 (50.0)	1 (25.0)	1 (33.3)	

Data are presented as median [interquartile range] for continuous variables and count (percentage) for categorical variables.

Abbreviations: FSGS, Focal segmental glomerulosclerosis; TIP, Tip variant; CEL, Cellular variant; NOS, Not otherwise specified; IQR, Inter quartile range; BP, Blood pressure; ACE-I, Angiotensin converting enzyme inhibitor; ARB, Angiotensin receptor blocker; eGFR, Estimate glomerular filtration rate.

### Pathological characteristics

Comparison of the pathological characteristics of the patients between the typical FSGS group and the unclassified group are shown in S2 Table. The pathological findings of the patients among the subgroups are also shown in Table 2. The timing of biopsy from the onset of disease and the interval between biopsy and the initiation of immunosuppressive treatment were not different among the subgroups. The number of total glomeruli and glomeruli with global sclerosis showed no significant difference among the subgroups. The median number of glomeruli with evaluated segmental lesions was 1 to 3 and the percentage of that was higher in the patients of CEL (17.2%) and minor cellular lesion (16.7%). The grade of IF/TA was higher in the patients of CEL and NOS, in whom more than 50% of the patients had interstitial and tubular damage in over 25% of the area.

**Table 2:** Pathological findings of the subgroups.

	Overall (n = 48)	Typical FSGS group			Unclassified group			P-value
		TIP (n = 13)	CEL (n = 15)	NOS (n = 6)	Endothelial damage (n = 6)	Simple attachment (n = 5)	Minor cellular lesion (n = 3)	
**Timing of biopsy**								
Disease duration until biopsy, months	2.0 [1.0–5.0]	1.0 [0.8–1.5]	3.0 [1.0–7.0]	1.5 [1.0–24.0]	3.5 [2.0–8.0]	2.0 [2.0–4.0]	1.0 [1.0–1.0]	0.107
IST start prior to biopsy	7 (14.6)	1 (7.7)	3 (20.0)	3 (50.0)	0 (0.0)	0 (0.0)	0 (0.0)	0.098
Time from biopsy to the initiation of IST, days	6 [1–21]	2 [1–14]	5 [2–9]	4 [-18-20]	27.5 [10–59]	9 [5–99]	13 [1–15]	0.28
**Pathological findings**								
Total glomeruli	24 [17–32]	32 [24–37]	21 [17–26]	18 [12–35]	29 [25–35]	17 [16–25]	18 [5–24]	0.112
Number of GS	1 [0–3]	1 [0–1]	2 [1–4]	2 [1–3]	3 [1–4]	2 [2–2]	0 [0–2]	0.26
Percentages of GS, %	5.8 [0.0–12.3]	2.7 [0.0–5.9]	7.4 [3.5–20.0]	10.7 [2.9–20.0]	12.7 [2.3–13.8]	11.8 [6.5–12.0]	0 [0.0–8.3]	0.152
Number of SL	2 [1–4]	2 [1–3]	3 [1–6]	3 [1–5]	1 [1–2]	1 [1–2]	2 [1–3]	0.162
Percentages of SL, %	6.9 [4.5–20.0]	5.9 [4.9–16.7]	17.2 [7.1–25.9]	11 [0.0–23.8]	4.6 [3.4–6.7]	6.3 [5.9–6.5]	16.7 [4.2–40.0]	0.029
Presence of findings relating to endothelial damage*	28 (58.3)	4 (30.8)	14 (93.3)	3 (50.6)	6 (100.0)	0 (0.0)	1 (12.5)	< 0.001
IF/TA								
0; absent or < 5%	14 (29.2)	7 (53.9)	2 (13.3)	0 (0.0)	4 (66.7)	1 (20.0)	0 (0.0)	0.028
1; 6–25%	17 (35.4)	5 (38.5)	3 (20.0)	2 (33.3)	1 (16.7)	3 (60.0)	3 (100.0)	
2; 26–50%	13 (27.1)	1 (7.7)	7 (46.5)	3 (50.0)	1 (16.7)	1 (20.0)	0 (0.0)	
3; > 50%	4 (8.3)	0 (0.0)	3 (20.0)	1 (16.7)	0 (0.0)	0 (0.0)	0 (0.0)	

Data are presented as median [interquartile range] for continuous variables and count (percentage) for categorical variables.

*Presence of findings relating to endothelial damage: Double contour of glomerular basement membrane, mesangiolysis with any degree.

Abbreviations: FSGS, Focal segmental glomerulosclerosis; TIP, Tip variant; CEL, Cellular variant; NOS, Not otherwise specified; IQR, Inter quartile range; IST, Immunosuppressive treatment; GS, Global sclerosis; SL, Segmental lesions; IF, Interstitial fibrosis; TA, Tubular atrophy.

### Therapeutic reactivity

**Fig 3:**
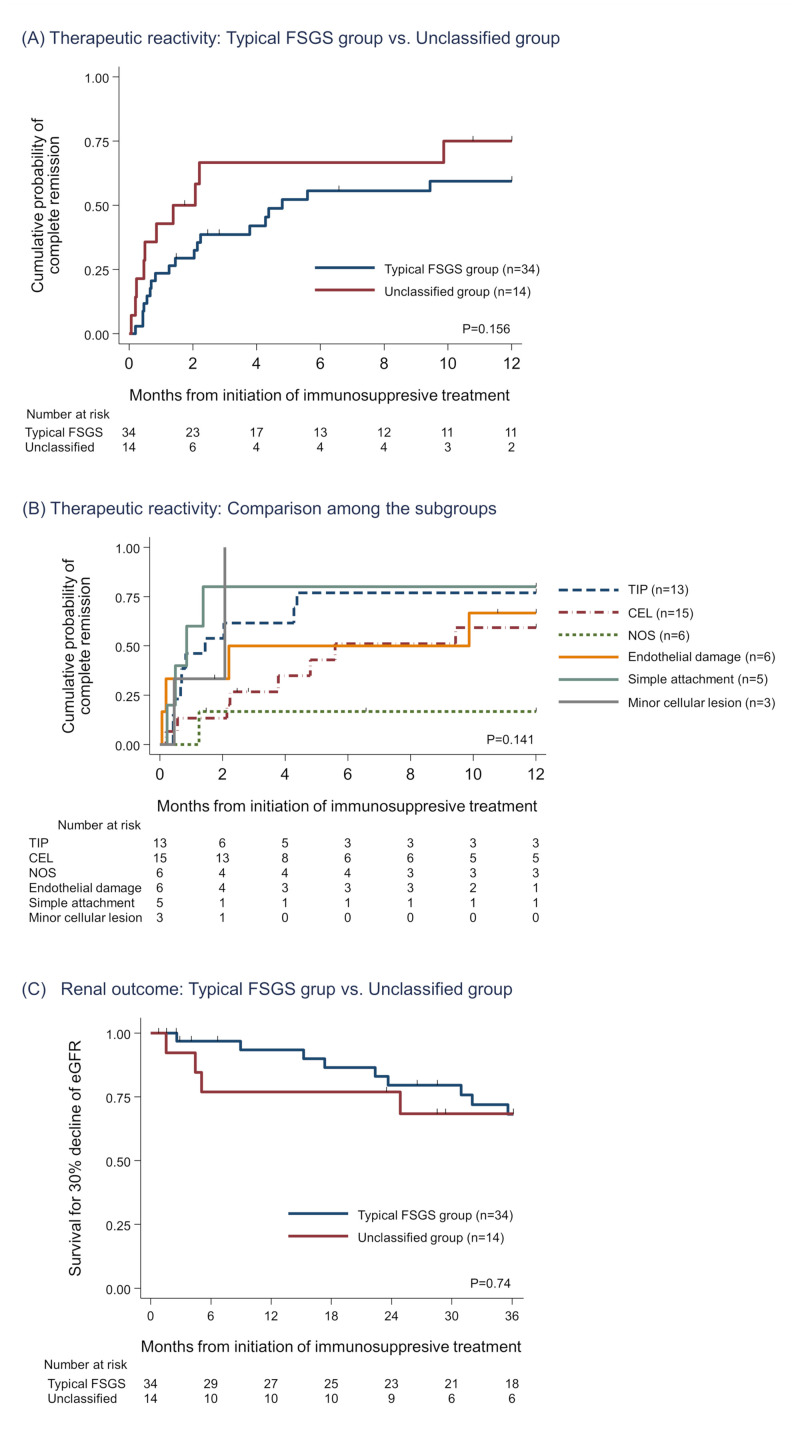
Outcome evaluation among typical variants of FSGS vs. unclassified group and their subgroups. (A and B) Kaplan-Meier analysis for cumulative probability of complete remission. There were no significant differences between the typical FSGS group and the unclassified group (A). Among the subgroups, TIP and “simple attachment” had the best therapeutic reactivity. CEL and “endothelial damage” showed moderate response to immunosuppressive treatment (B). (C) Kaplan-Meier curve for 30% decline of eGFR. No significant differences were observed in comparison between the typical FSGS group and the unclassified group and comparison among the subgroups (S2 Fig). Abbreviations: FSGS, Focal segmental glomerulosclerosis; CEL, Cellular variant; TIP, Tip variant; NOS, Not otherwise specified; eGFR, Estimated glomerular filtration ratio.

All patients received immunosuppressive treatment, and details of them and outcomes of the patients are summarized in comparison between the typical FSGS and the unclassified group (S3 Table) and among the subgroups (Table 3). There were no differences in the treatment types. During entire observation (median: 49.4 months, 1–147 months), the overall probability of CR was 68.8% (TIP, 84.6%; CEL, 60.0%; NOS, 50.0%; endothelial damage, 66.7%; simple attachment 40.0%; and minor cellular lesions, 66.7%; P = 0.65). Comparing the time to CR from the baseline, there was no significant differences between the typical FSGS group and the unclassified group (Fig 3A). Among the subgroups, TIP showed the best response, CEL demonstrated a moderate response, and NOS showed the worst response among the patients with typical FSGS variants. All patients except one in the “simple attachment” subgroup showed an excellent response to the initial treatment. Although the number of patients was limited, the patients of “minor cellular lesion” subgroup demonstrated good response following to “simple attachment”. Whereas, patients in the “endothelial damage” subgroup demonstrated a moderate response similar to CEL (Fig 3B). As secondary outcomes for treatment response, the percentages of the patients who attained partial remission and non-response were 95.8% and 4.2% respectively. No difference was observed in these secondary outcomes among the subgroups.

**Table 3:** Details of immunosuppressive treatment and outcomes of the subgroups.

	Overall (n = 48)	Typical FSGS group			Unclassified group			P-value
		TIP (n = 13)	CEL (n = 15)	NOS (n = 6)	Endothelial damage (n = 6)	Simple attachment (n = 5)	Minor cellular lesion (n = 3)	
**Follow-up**								
Entire observation, months	49.4 [24.3–75.3]	63.4 [48.7–75.6]	41.5 [14.4–80.2]	60.7 [6.6–78.9]	27.2 [23.5–44.4]	56.0 [28.5–60.1]	1.7 [0.7–95.5]	0.34
**Details of treatment**								
Initial PSL dose, mg/day	47.5 [40–52.5]	50 [40–55]	50 [40–60]	42.5 [40–50]	35 [30–50]	40 [40–60]	40 [40–50]	0.66
Initial PSL dose, mg/kg/day	0.73 [0.64–0.87]	0.71 [0.61–0.93]	0.69 [0.63–0.85]	0.73 [0.63–0.90]	0.76 [0.73–0.81]	0.75 [0.64–0.79]	0.82 [0.68–0.94]	0.98
mPSL pulse therapy	10 (20.8)	4 (30.8)	3 (20.0)	2 (33.3)	0 (0.0)	1 (20.0)	0 (0.0)	0.59
Use of ISAs	19 (39.6)	4 (30.8)	8 (53.3)	3 (50.0)	3 (50.0)	1 (20.0)	0 (0.0)	0.43
ISA detail		CyA (4)	CyA (7), MZR (1)	CyA (3)	CyA (3)	CyA (1)		
Days to start ISA, days	17 [6–40]	6.5 [3–214]	22 [9–31.5]	26 [14–77]	14 [0–140]	52 [52–52]	-	0.70
Intravenous 25% albumin	5 (10.4)	1 (7.7)	2 (13.3)	0 (0.0)	0 (0.0)	1 (20.0)	1 (33.3)	0.58
LDL-apheresis	8 (16.7)	1 (7.7)	3 (20.0)	1 (16.7)	1 (16.7)	2 (40.0)	0 (0.0)	0.63
Plasma exchange	1 (1.7)	0 (0.0)	1 (6.6)	0 (0.0)	0 (0.0)	0 (0.0)	0 (0.0)	-
**Outcomes**								
Complete remission	33 (68.8)	11 (84.6)	9 (60.0)	3 (50.0)	4 (66.7)	4 (80.0)	2 (66.7)	0.65
Days to complete remission	44 [15–133]	25 [14–130]	115 [65–170]	574 [38–1830]	37 [4–184]	20.5 [11–34]	38.5 [14–63]	0.21
Partial remission	46 (95.8)	13 (100.0)	14 (93.3)	5 (83.3)	6 (100.0)	5 (100.0)	3 (100.0)	0.59
Non-response	2 (4.2)	0 (0.0)	1 (6.7)	1 (16.7)	0 (0.0)	0 (0.0)	0 (0.0)	0.59
Death	4 (8.7)	0 (0.0)	2 (14.3)	0 (0.0)	0 (0.0)	1 (20.0)	1 (33.0)	0.26
30% decline of eGFR	14 (29.8)	0 (0.0)	7 (46.7)	5 (60.0)	2 (33.3)	1 (20.0)	1 (33.0)	0.105
Maintenance dialysis	5 (10.4)	0 (0.0)	4 (26.7)	0 (0.0)	1 (16.7)	0 (0.0)	0 (0.0)	0.178
Kidneny transplantation	0 (0.0)	0 (0.0)	0 (0.0)	0 (0.0)	0 (0.0)	0 (0.0)	0 (0.0)	-

Data are presented as median [interquartile range] for continuous variables and count (percentage) for categorical variables.

Definitions: Complete remission, reduction of proteinuria to < 0.3 g/day or < 0.3 g/gCr; Partial remission, reduction of proteinuria to < 3.5 g/day or < 3.5 g/gCr; Non-response, failed to reduce urine protein less than 3.5 g/day or 3.5 g/gCr.

Abbreviations: FSGS, Focal segmental glomerulosclerosis; TIP, Tip variant; CEL, Cellular variant; NOS, Not otherwise specified; IQR, Inter quartile range; mPSL, Methylprednisolone; PSL, Prednisolone; ISA, Non-steroidal immunosuppressive agent; CyA, Cyclosporine; MZR, Mizoribine; LDL, Low density lipoprotein; eGFR, Estimate glomerular filtration rate.

### Renal outcome

In the initial phase of the treatment, 5 (10.4%) patients received temporary hemodialysis due to volume overload or acute kidney injury (Table 1), and all of them recovered and withdrew dialysis within 2 months. During the observation period, 14 (29.8%) patients experienced 30% decrease of eGFR, and 5 (10.4%) patients finally required maintenance dialysis therapy, but none of the patients in the cohort received kidney transplantation (Table 3, S3 Table). A Kaplan-Meier curve for 30% decline of eGFR showed that no significant differences were observed not only between the typical FSGS group and the unclassified group (Fig 3C) but also among the subgroups (S2 Fig). However it was difficult to compare each subgroup because of the limited sample size and number of the outcome.

## Discussion

FSGS reveals variety of glomerular lesions: Classical sclerosis or typical variants of the Columbia classification. However, we occasionally encounter segmental glomerular lesions which does not meet the criteria the Columbia classification, in practice. The present study is the first to evaluate the clinical implications of such unclassified glomerular lesions.

Compared with previous studies, our study has a number of strengths. First, the subjects of our study were limited to adult patients with NS. Since FSGS patients are clinically heterogenous with varying etiologies [1,20,21], treatment strategies are depended on clinical status of the patients and immunosuppressive treatments are mainly prescribed to the cases with NS. Therefore, it is particularly important to address the patients’ relevance, including their background, when discussing the differences among histopathological subgroups of FSGS patients [22]. However, most of previous studies mixed adults and children, and included non-nephrotic patients who did not receive immunosuppressive treatment. In order to evaluate the association between pathological findings and their treatment response, we enrolled adult treatment-naïve NS patients who were considered as primary forms. Additionally, the patients were treated basically by the one principle of our institutes with common therapeutic regimen. Second, reevaluation of the pathology was carried out using the many serial sections with different staining, in order to trace the atypical segmental lesion in a single slide that might be a part of typical segmental lesions. Furthermore, all the pathological slides and paraffin blocks, except one, were prepared and stored in a single institute; this provided us with uniform pathological samples for review. With these advantages, the present study comprehensively addressed the significance of the abovementioned unclassified lesions.

### General characteristics of the cohort

In the present study, 172 out of 5870 patients (2.9%) were eligible for the evaluation of segmental lesions. A Japanese nation-wide registry study reported that the prevalence of FSGS was 3–4% of the total kidney biopsy in Japan [23]. Although the number of the patients in our study was small, this prevalence was almost consistent with the nation-wide data.

In previous studies, the most prevalent variant was NOS followed by PH or COL [10–15]. In particular, CEL are rare in the Western cohorts and the clinical importance of this variant is not well demonstrated. Whereas, TIP and CEL were the most common in our cohort, and there were no COL or PH patients. This unique distribution could be derived from our strict inclusion criteria and homogenous ethnicity of Japanese participants. In our cohort, 68.8% of the patients attained CR, which was similar to other Japanese cohort studies [14,24] and better comparing with previous studies from other countries [9–13].

### Unclassified lesions: Comparison with typical FSGS or MCD

In current practice, it is difficult to make a decision to diagnose FSGS or MCD when the case does not fit the Columbia classification. The unclassified lesions that we evaluated have not been classified into MCD nor FSGS even as NOS, which is originally defined as “focal and segmental consolidation of the tuft by increased extracellular matrix, obliterating the glomerular capillary lumen/lumina” after excluding other subtypes of variants [8]. In pathological evaluation, the frequency of the glomeruli with segmental lesions in unclassified group was low (6.1%), which was not different from that in typical FSGS group (10.6%) (P = 0.093, S2 Table). In clinical evaluation, cumulative probability of CR at 12 months in typical FSGS and unclassified group was 0.59 [95% confidence interval (95%CI): 0.42, 0.77] and 0.75 [95%CI: 0.50, 0.94], respectively (S3 Table). A Japanese nationwide cohort study reported that the cumulative probability of CR at 12 months form the baseline for FSGS and MCD was 0.61 [95%CI: 0.41. 0.74] and 0.93 [95%CI: 0.87, 0.96], respectively [24]. Therefore, the cases with unclassified lesions were considered not as MCD accompanied with these lesions by chance, but as different patient group from MCD with moderate treatment response.

### Clinical significance of endothelial damage

In our results, the patients of “endothelial damage” subgroup showed a similar clinical course to CEL. This subgroup was characterized by segmental mesangiolysis and double contour appearance of GBM. Segmental mesangiolysis and double contour appearance of GBM are both caused by endothelial injury [25–27]. Although FSGS is considered to be on the spectrum of podocyte injury [1,21,28], glomerular endothelial injury is also associated with the progression of FSGS lesions. Previous study using a podocyte selective injury model of collapsing FSGS suggested that podocyte injury causes endothelial damage by local crosstalk signaling [29]. Podocyte is major source of vascular endothelial growth factor (VEGF), and anti-VEGF antibody, anti-VEGF ligand, and receptor tyrosine kinase inhibitors similarly caused heavy proteinuria with TMA and FSGS lesions [30,31]. Several reports described the overlap between disorders with endothelial damage and histological FSGS: Preeclampsia [32], TMA induced by malignant hypertension [33] or reno-vascular hypertension [34]. A retrospective study showed that FSGS, particularly COL, was commonly (62.3%) observed among cases with TMA [35]. In our study, more than 50% of CEL variants contained lesion with endothelial damage (Table 2). Together, these findings suggest that TMA-like lesions may likely be incipient or precursor lesions of the typical FSGS associated with podocyte-injury.

### Clinical significance of simple attachment

Our results showed that the subgroup of “simple attachment” demonstrated a similar clinical course to TIP or CEL. Glomerular synechiae, an attachment between the glomerular tuft and the Bowman’s capsule, represents an initial lesion in post-adaptive FSGS [6,36]. The terminology of synechiae is ambiguous because there is no detailed definition regarding the degree of adhesion. It describes that “the smallest FSGS lesions may consist of a simple synechial attachment, without prominent matrix accumulation in the underlying glomerular tuft” [37]. Smeets et al. experimentally showed that parietal epithelial cells (PECs) play an essential role in forming bridges between the tuft and the Bowman’s capsule and development of sclerosing lesion in the FSGS model [38]. They reported that activated PEC, which stained positively for CD44, could guide to discriminate early FSGS from MCD on human renal biopsy specimens [39]. Therefore, the “simple attachment” could indicate the earliest change of some lesions of FSGS occurring by interaction between the podocytes and the PECs.

### Clinical significance of minor cellular lesions

Although glomerular foam cell, that is promoted by podocyte injury [40], has diagnostic value for CEL, it can also be observed in other variants of FSGS by Columbia classification. CEL is defined as an endocapillary hypercellularity that involves at least 25% of the tuft [8]. If the patients only have a cellular lesion that involves less than 25% of the tuft, despite serial section analysis, they cannot be classified as any of the variants. It remains unclear whether the minor accumulation of foam cell(s) indicates a precursor lesion of typical CEL or occasionally reversible. Although the sample size was quite limited in our study, the patients in this subgroup demonstrated better therapeutic reactivity compared to those with typical TIP or CEL. Further clinical investigations with a larger sample size are required.

Our results suggested that three type of unclassified lesions that we evaluated were clinically equivalent to typical FSGS. In addition, these unclassified lesions were inferred to associate with the typical FSGS lesions in the Columbia classification including the possibility as precursor findings of them. In order to investigate the relationship between the unclassified findings and typical lesions, experimental validation on maturing process of FSGS lesion are also required.

We should mention the limitations of this study. This is a retrospective study with a limited sample size among homogenous Japanese patients. Particularly, the number of patients with minor cellular lesion (n = 3) was not sufficient. Therefore, further studies with a larger sample size and multi-ethnical participants are needed to determine the clinical relevance of unclassified FSGS lesions.

In conclusion, nephrotic patients with the unclassified segmental glomerular lesions that do not fit the Columbia classification: endothelial damage, simple attachment and minor cellular lesion could be referred as FSGS.

## Supporting information

S1 Fig Pathological findings of the patients in the unclassified group (additional images). (PDF)

S2 Fig Kaplan-Meier curve for 30% decline of eGFR among the subgroups. (PDF)

S1 Table Baseline characteristics of the typical FSGS group and the unclassified group. (PDF)

S2 Table Pathological findings of the typical FSGS group and the unclassified group. (PDF)

S3 Table Details of immunosuppressive treatment and outcomes of the typical FSGS group and the unclassified group. (PDF)
