# Supplementary material for: Nephrotic syndrome with focal segmental glomerular lesions unclassified by Columbia classification; Pathology and clinical implication
Source: PLoS One. 2021 Jan 5;16(1):e0244677. doi: 10.1371/journal.pone.0244677 (PMC7785116; doi:10.1371/journal.pone.0244677)
Supplement: S1 Fig — (PDF) [file pone.0244677.s001.pdf]

S1 Fig. Pathological findings of the patients in the unclassified group (additional images)

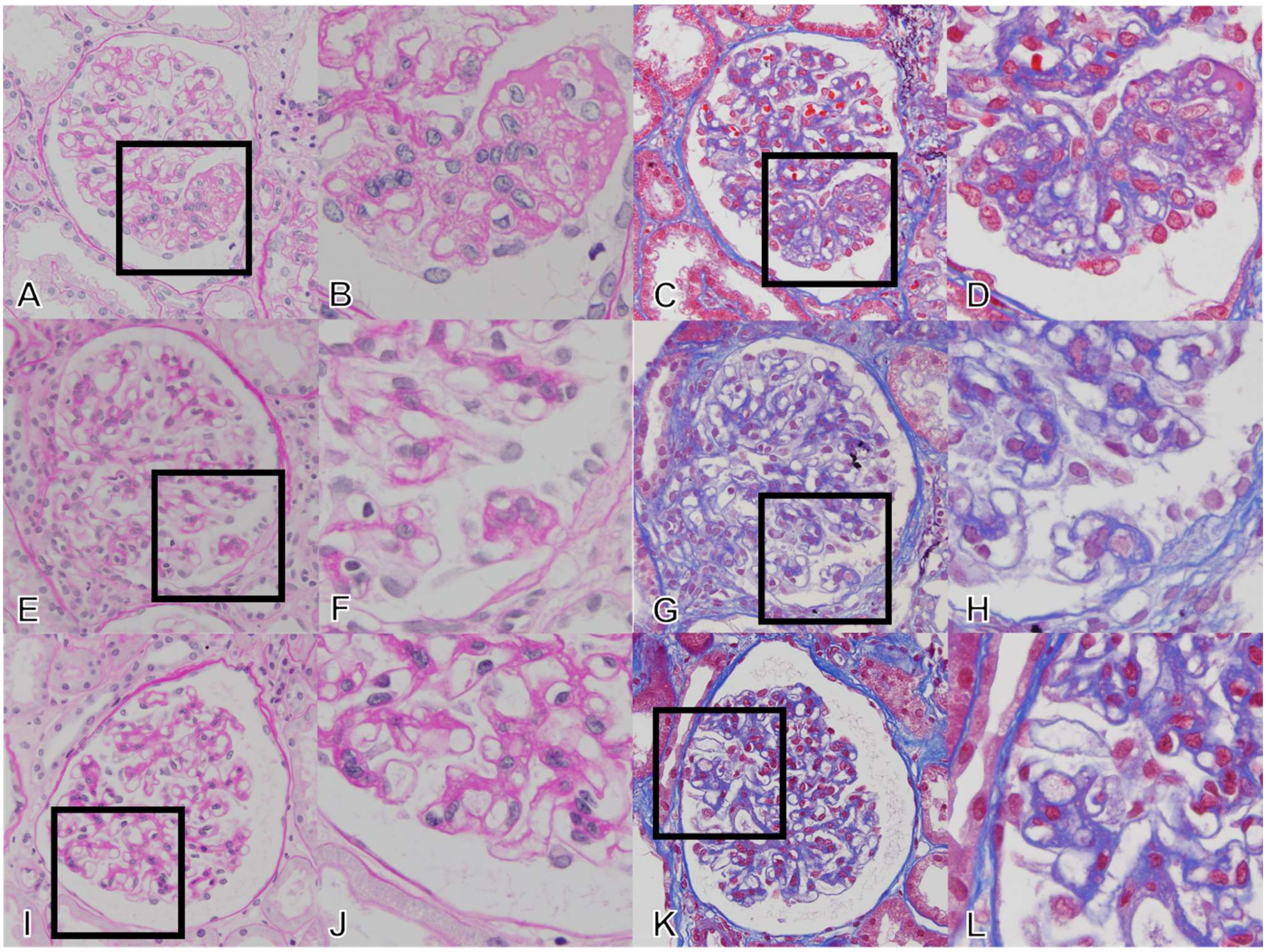

Typical finding of “endothelial damage” by PAS stain (A and B) and Masson trichrome stains (C and D).
The finding of “simple attachment” by PAS stain (E and F) and Masson trichrome stains (G and H).
The finding of “minor cellular lesion” by PAS stain (I and J) and Masson trichrome stains (K and L).
The patients had no other findings on typical FSGS.
A, E, and I are at ×400 magnification with PAS staining, and B, F, and J are at ×1000 magnification with PAS staining. C, G, and K are at ×400 magnification with Masson trichrome stains, and B, F, and J are at ×1000 magnification with Masson trichrome stains.

Abbreviations: FSGS, Focal segmental glomerulosclerosis; PAS, Periodic acid Schiff stain
