# Supplementary material for: Nephrotic syndrome with focal segmental glomerular lesions unclassified by Columbia classification; Pathology and clinical implication
Source: PLoS One. 2021 Jan 5;16(1):e0244677. doi: 10.1371/journal.pone.0244677 (PMC7785116; doi:10.1371/journal.pone.0244677)
Supplement: S2 Fig — (PDF) [file pone.0244677.s002.pdf]

S2 Fig. Kaplan-Meier curve for 30% decline of eGFR among the subgroups

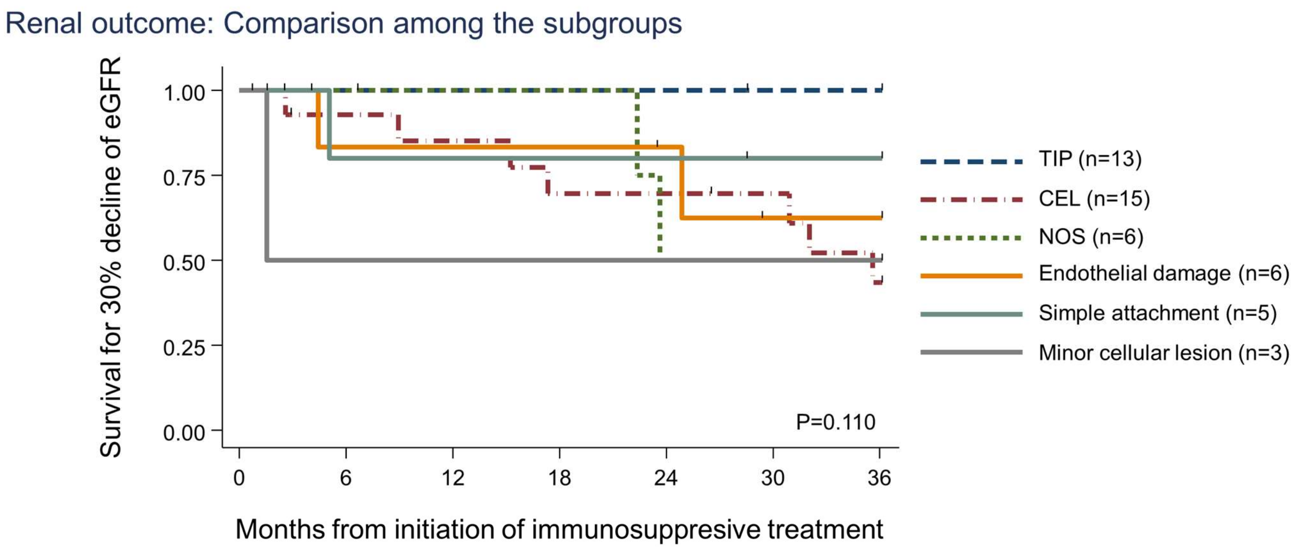

Number at risk							
TIP	13	12	12	12	12	11	11
CEL	15	12	11	9	9	8	5
NOS	6	5	4	4	2	2	2
Endothelial damage	6	5	5	5	4	2	2
Simple attachment	5	4	4	4	4	3	3
Minor cellular lesion	3	1	1	1	1	1	1

No significant differences were observed in comparison among the subgroups. However, because of the limited sample size and number of the outcome, it was difficult to compare each subgroup.

Abbreviations: FSGS, Focal segmental glomerulosclerosis; CEL, Cellular variant; TIP, Tip variant; NOS, Not otherwise specified; eGFR, Estimated glomerular filtration ratio
