# Supplementary material for: Nephrotic syndrome with focal segmental glomerular lesions unclassified by Columbia classification; Pathology and clinical implication
Source: PLoS One. 2021 Jan 5;16(1):e0244677. doi: 10.1371/journal.pone.0244677 (PMC7785116; doi:10.1371/journal.pone.0244677)
Supplement: S1 Table — (PDF) [file pone.0244677.s003.pdf]

S1 Table. Baseline characteristics of the typical FSGS group and the unclassified group

	Typical FSGS group (n=34)		Unclassified group (n=14)		P-value
**Patient characteristics**					
Age	56	[39-66]	65	[60-72]	0.013
Sex, male	25	(73.5)	8	(57.1)	0.27
Height, cm	165.0	[158.2-170.0]	159.3	[155.0-165.0]	0.134
Body weight, kg	63.7	[57.8-71.7]	58.3	[53.6-62.9]	0.107
Body mass index	23.5	[21.8-26.3]	23.1	[21.0-24.6]	0.31
Systolic BP, mmHg	142	[122-150]	123	[119-141]	0.051
Diastolic BP, mmHg	81	[73-90]	78	[66-84]	0.097
Diabetes mellitus	1	(2.9)	1	(7.1)	0.51
Hypertension	14	(41.2)	5	(35.7)	0.73
ACE-I/ARB	13	(38.2)	6	(42.9)	0.77
Diuretics	20	(58.8)	10	(71.4)	0.41
Statins	15	(44.1)	7	(50.0)	0.71
**Laboratory data**					
Total protein, g/dL	4.5	[3.7-4.9]	4.9	[3.7-5.4]	0.43
Albumin, g/dL	1.9	[1.5-2.2]	2.1	[1.5-2.3]	0.30
Creatinine, mg/dL	1.10	[0.86-1.68]	0.90	[0.74-1.44]	0.163
eGFR, mL/min/1.73m^2	50.9	[37.9-69.4]	62.0	[38.1-71.5]	0.62
Temporary dialysis	4	(11.8)	1	(7.1)	0.63
Urinary protein, g/gCr	7.43	[5.06-9.41]	6.58	[5.51-7.79]	0.35
Urinary occult blood					
(-)	3	(8.8)	1	(7.7)	
(+/-)	1	(2.9)	1	(7.7)	
(1+)	12	(35.3)	3	(23.1)	0.54
(2+)	12	(35.3)	3	(23.1)	
(3+)	6	(17.7)	5	(38.5)	

Data are presented as median [interquartile range] for continuous variables and count (percentage) for categorical variables.

Abbreviations: FSGS, Focal segmental glomerulosclerosis; TIP, Tip variant; CEL, Cellular variant; NOS, Not otherwise specified; IQR, Inter quartile range; BP, Blood pressure; ACE-I, Angiotensin converting enzyme inhibitor; ARB, Angiotensin receptor blocker; eGFR, Estimate glomerular filtration rate
