# Supplementary material for: Nephrotic syndrome with focal segmental glomerular lesions unclassified by Columbia classification; Pathology and clinical implication
Source: PLoS One. 2021 Jan 5;16(1):e0244677. doi: 10.1371/journal.pone.0244677 (PMC7785116; doi:10.1371/journal.pone.0244677)
Supplement: S2 Table — (PDF) [file pone.0244677.s004.pdf]

S2 Table. Pathological findings of the typical FSGS group and the unclassified group

	Typical FSGS group (n=34)		Unclassified group (n=14)		P-value
Disease duration until biopsy, months	1.5	[1.0-5.0]	2.0	[1.0-6.0]	0.47
Use of immunosuppressive drugs prior to biopsy	7	(20.6)	0	(0.0)	0.066
Time from biopsy to the initiation of immunosuppressive treatment, days	4	[1-14]	14	[5-59]	0.020
**Pathological findings**					
Total glomeruli	24	[17-34]	25	[16-29]	0.64
Number of GS	1	[0-2]	2	[0-3]	0.57
Percentages of GS, %	5.3	[0.0-8.3]	10.0	[0.0-12.5]	0.65
Number of SL	2	[1-5]	1	[1-2]	0.017
Percentages of SL, %	10.6	[4.9-16.7]	6.1	[4.0-8.0]	0.093
Presence of findings relating to endothelial damage*, %	21	(61.8)	7	(50.0)	0.45
IF/TA					
0; absent or < 5%	9	(26.5)	5	(35.7)	0.23
1; 6–25%	10	(29.4)	7	(50.0)	
2; 26–50%	11	(32.4)	2	(14.3)	
3; > 50%	4	(11.8)	0	(0.0)	

Data are presented as median [interquartile range] for continuous variables and count (percentage) for categorical variables.

*Presence of findings relating to endothelial damage: Double contour of glomerular basement membrane, mesangiolysis with any degree.

Abbreviations: FSGS, Focal segmental glomerulosclerosis; TIP, Tip variant; CEL, Cellular variant; NOS, Not otherwise specified; IQR, Inter quartile range; GS, Global sclerosis; SL, Segmental lesions; IF, Interstitial fibrosis; TA, Tubular atrophy
