# Supplementary material for: Nephrotic syndrome with focal segmental glomerular lesions unclassified by Columbia classification; Pathology and clinical implication
Source: PLoS One. 2021 Jan 5;16(1):e0244677. doi: 10.1371/journal.pone.0244677 (PMC7785116; doi:10.1371/journal.pone.0244677)
Supplement: S3 Table — (PDF) [file pone.0244677.s005.pdf]

S3 Table. Details of immunosuppressive treatment and outcomes of the typical FSGS group and the unclassified group

	Typical FSGS group (n=34)		Unclassified group (n=14)		P-value
**Follow-up**					
Entire observation, months	60.9	[28.5-78.9]	28.9	[18.45-56.0]	0.049
**Details of treatment**					
Initial PSL dose, mg/day	50	[40-55]	40	[40-50]	0.180
Initial PSL dose, mg/kg/day	0.70	[0.63-0.90]	0.76	[0.68-0.82]	0.79
mPSL pulse therapy	9	(26.5)	1	(7.1)	0.134
Use of ISAs	15	(44.1)	4	(28.6)	0.32
ISA detail	CyA (14), MZR(1)		CyA (4)		
Days to start ISA, days	17	[6-35]	33	[7-96]	0.65
Intravenous 25% albumin	3	(8.8)	2	(14.3)	0.57
LDL-apheresis	5	(14.7)	3	(21.4)	0.57
Plasma exchange	1	(2.9)	0	(0.0)	-
**Outcomes**					
Complete remission	23	(67.6)	10	(71.4)	0.80
Days to complete remission	65	[20-170]	21	[7-63]	0.066
Cumulative probability for CR [95%CI]					
1 month	0.24	[0.13, 0.42]	0.43	[0.22, 0.72]	
2 months	0.29	[0.17, 0.48]	0.50	[0.28, 0.77]	
6 months	0.56	[0.39, 0.73]	0.67	[0.42, 0.89]	
12 months	0.59	[0.42, 0.77]	0.75	[0.50, 0.94]	
Partial remission	32	(94.1)	14	(100.0)	0.35
Non-response	2	(5.9)	0	(0.0)	0.35
Death	3	(8.8)	2	(14.3)	0.62
30% decline of eGFR	10	(29.4)	4	(28.6)	0.95
Maintenance dialysis	4	(11.8)	1	(7.1)	0.63
Kidney transplantation	0	(0.0)	0	(0.0)	-

Data are presented as median [interquartile range] for continuous variables and count (percentage) for categorical variables.

Definitions: Complete remission, reduction of proteinuria to < 0.3 g/day or < 0.3 g/gCr; Partial remission, reduction of proteinuria to < 3.5 g/day or < 3.5 g/gCr; Non-response, failed to reduce urine protein less than 3.5 g/day or 3.5 g/gCr.

Abbreviations: FSGS, Focal segmental glomerulosclerosis; TIP, Tip variant; CEL, Cellular variant; NOS, Not otherwise specified; IQR, Inter quartile range; mPSL, Methylprednisolone; PSL, Prednisolone; ISA, Non-steroidal immunosuppressive agent; CyA, Cyclosporine; MZR, Mizoribine; LDL, Low density lipoprotein; CR, Complete remission; eGFR, Estimate glomerular filtration rate
